# Supplementary figures and images for: Right ventricular pressure–volume relations and effects of selective vena cava occlusion during cardiopulmonary resuscitation
Source: PLoS One. 2025 Sep 26;20(9):e0333122. doi: 10.1371/journal.pone.0333122 (PMC12469095; doi:10.1371/journal.pone.0333122)

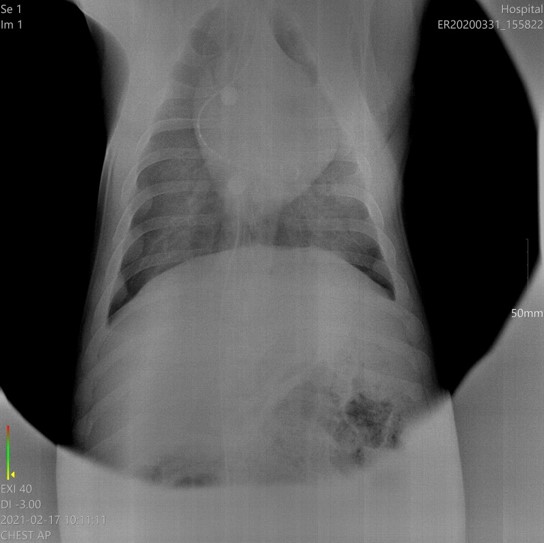

Supplement: S1 Fig — Chest anteroposterior X-ray image showing catheter placement. The image was obtained using a portable chest X-ray device. The red arrow indicates a PV catheter in the right ventricle. The blue arrows indicate the balloon catheters in the proximal portion of the SVC and IVC. PV, pressure–volume; SVC, superior vena cava; IVC, inferior vena cava. (DOCX) [file pone.0333122.s001.docx]

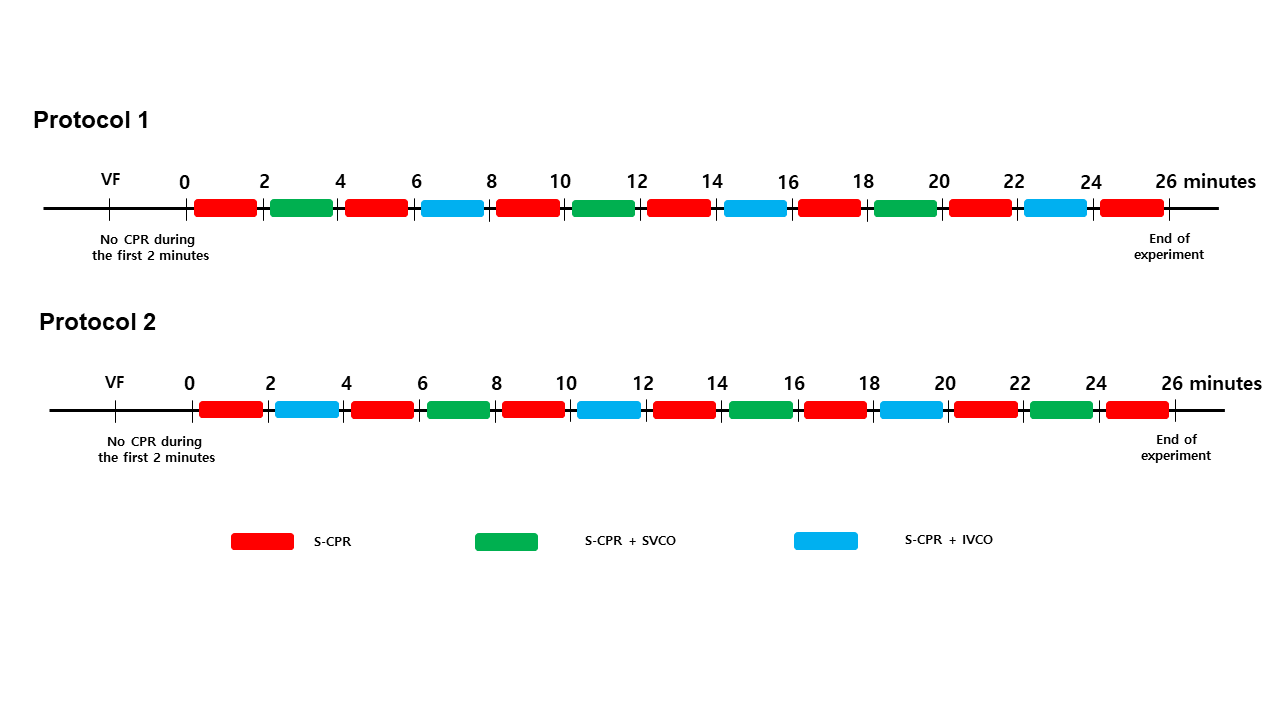

Supplement: S2 Fig — The experimental protocols used during CPR to assess the hemodynamic effects of VCO. Two protocols were applied to minimize time-dependent variations. In Protocol 1, 2-min periods of CPR with No-VCO, SVCO, No-VCO, and IVCO were sequentially performed. In Protocol 2, the sequence was No-VCO, IVCO, No-VCO, and SVCO, for 2 min each. The sequence was repeated three times. VF, ventricular fibrillation; S-CPR, standard cardiopulmonary resuscitation with no vena cava occlusion; S-CPR + SVCO, standard cardiopulmonary resuscitation with superior vena cava occlusion, S-CPR + IVCO, standard cardiopulmonary resuscitation with inferior vena cava occlusion. (TIF) [file pone.0333122.s002.tif]

(A)


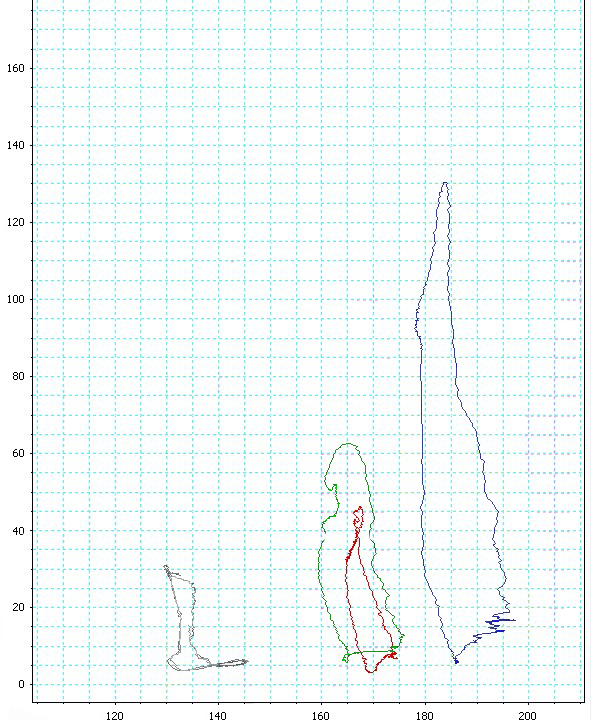


(B)


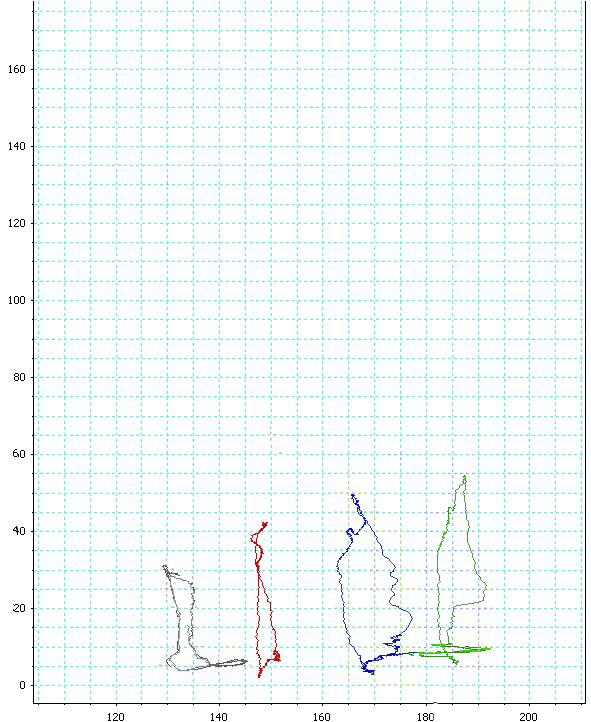


(C)


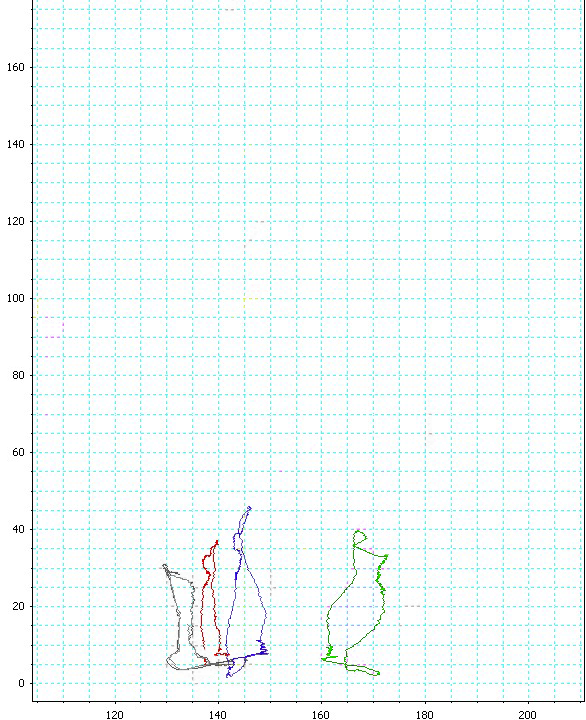

Supplement: S3 Fig — Measurement loops for the No-VCO, SVCO, and IVCO periods are shown in blue, green, and red, respectively. Compared to the spontaneous circulation period, marked in black, when CPR is performed with No-VCO, both RVVed and RVVes increase, but the effective volume does not seem to increase significantly. In addition, no significant differences were observed in the RV stroke volume between the No-VCO, SVCO, and IVCO conditions. RV PV, right ventricular pressure–volume; CPR, cardiopulmonary resuscitation; VCO, vena cava occlusion; SVCO, superior vena cava occlusion; IVCO, inferior vena cava occlusion; RVVed, end-diastolic right ventricular volume; RVVes, end-systolic right ventricular volume. (DOCX) [file pone.0333122.s003.docx]

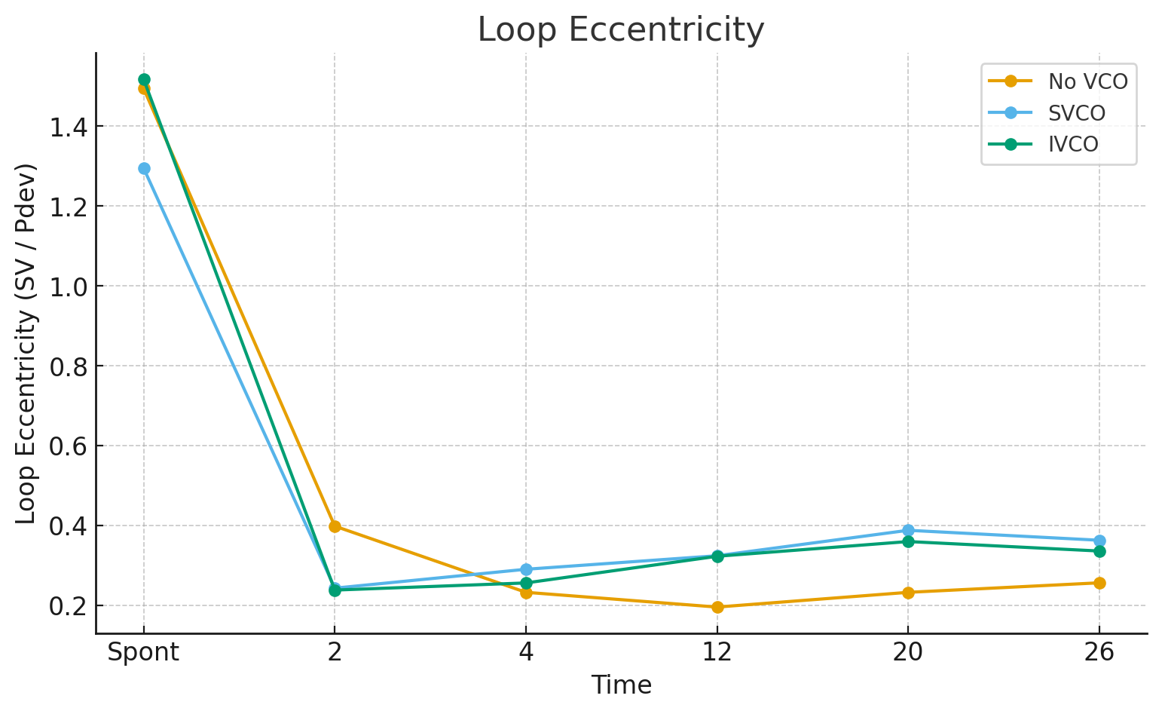

Supplement: S4 Fig — Measurement loops for the No-VCO, SVCO, and IVCO periods are shown in yellow, blue, and green, respectively. Loop eccentricity was calculated as (RVPes - RVPed)/ (RVVed - RVVes), where higher values indicates a more elongated loop, reflecting changes in RV loading conditions or systolic/diastolic imbalance. The eccentricity decreases over time, reflecting that the loop became relatively wider horizontally (greater volumetric than pressure fluctuations). RV PV, right ventricular pressure–volume; VCO, vena cava occlusion; SVCO, superior vena cava occlusion; IVCO, inferior vena cava occlusion; SV, right ventricular stroke volume; Pedv, developed pressure (maximum pressure minus minimal pressure); Spont; spontaneous circulation period. (TIF) [file pone.0333122.s004.tif]

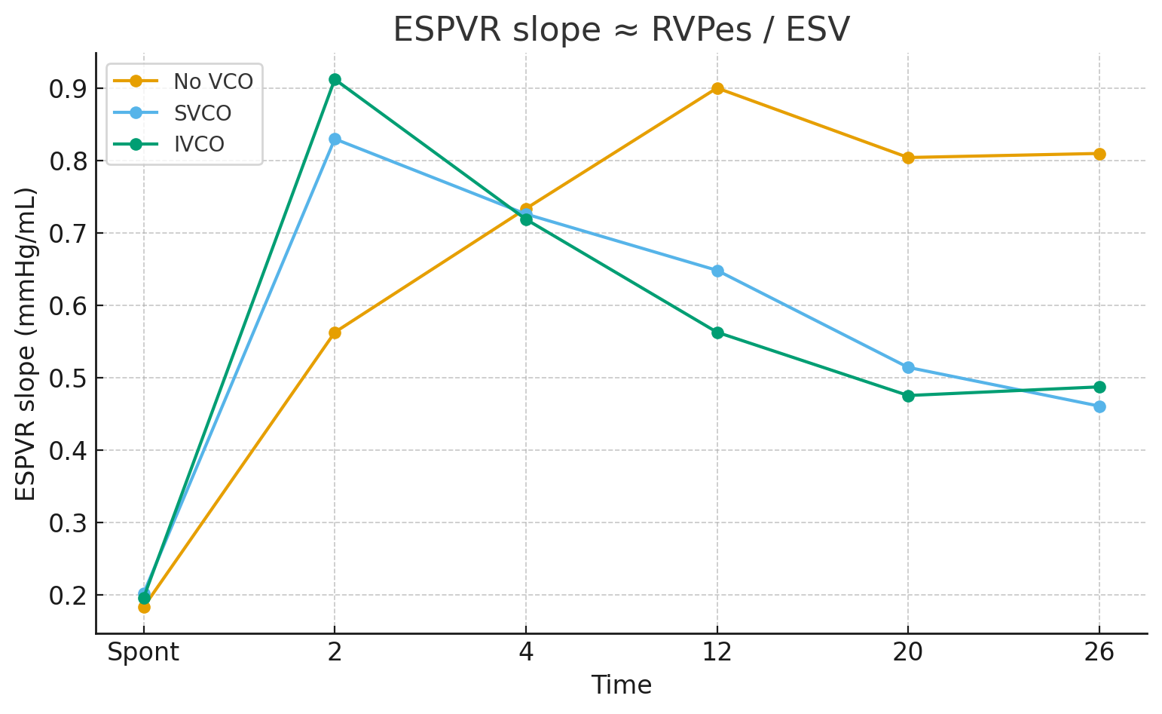

Supplement: S5 Fig — ESPVR was calculated as RVPes/ (RVVes - V0) and was used as an index of RV contractility. The ESPVR slope gradually decreased (decreased contractility) over the course of CPR in all three groups, with the lowest value occurring in the period of spontaneous circulation. ESPVR, end-systolic pressure–volume relationship; VCO, vena cava occlusion; SVCO, superior vena cava occlusion; IVCO, inferior vena cava occlusion; RVPes, end-systolic right ventricular pressure; ESV, end-systolic right ventricular volume; Spont; spontaneous circulation period. (TIF) [file pone.0333122.s005.tif]

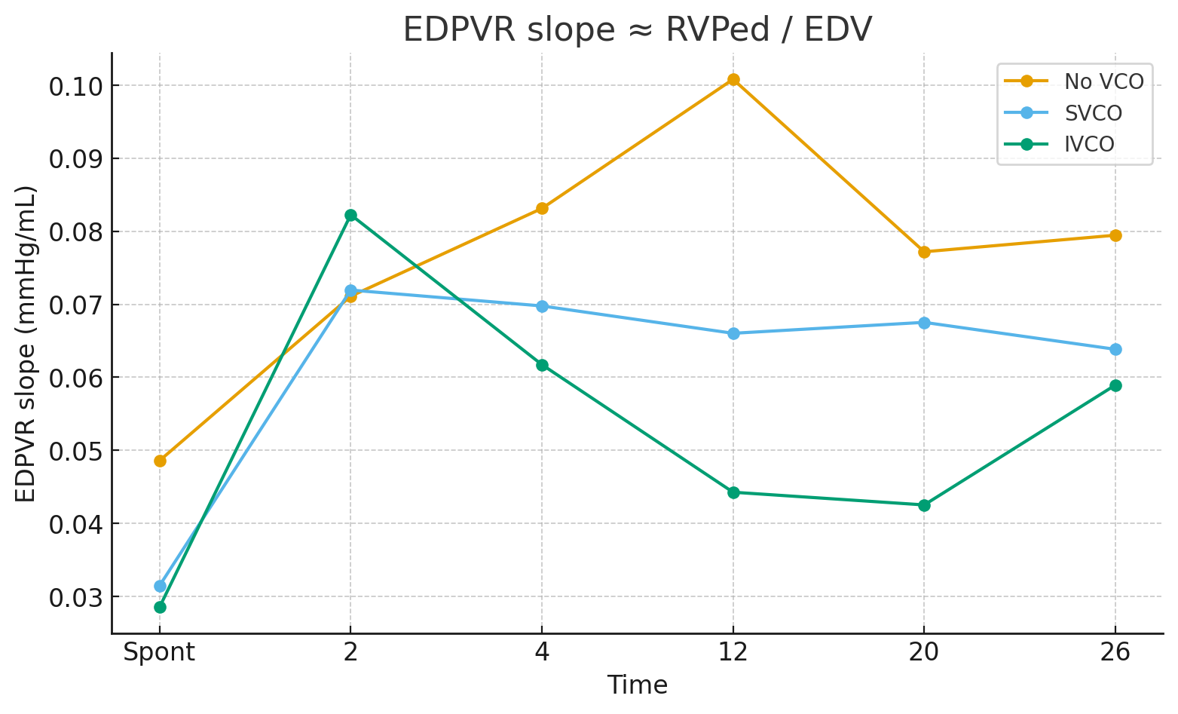

Supplement: S6 Fig — EDPVR was defined as RVPed/ RVVed and was used as an index of diastolic stiffness, with higher values indicating reduced compliance. The EDPVR slope remains low overall and shows only modest changes over time and across groups. EDPVR, end-diastolic pressure–volume relationship; VCO, vena cava occlusion; SVCO, superior vena cava occlusion; IVCO, inferior vena cava occlusion; RVPed, end-diastolic right ventricular pressure; EDV, end-diastolic right ventricular volume; Spont; spontaneous circulation period. (TIF) [file pone.0333122.s006.tif]
