# Supplementary material for: Right ventricular pressure–volume relations and effects of selective vena cava occlusion during cardiopulmonary resuscitation
Source: PLoS One. 2025 Sep 26;20(9):e0333122. doi: 10.1371/journal.pone.0333122 (PMC12469095; doi:10.1371/journal.pone.0333122)
Supplement: S1 Table — (DOCX) [file pone.0333122.s007.docx]

**S1 Table.** Baseline characteristics and measurements of animals included in Experiments I and II.

| Parameters | Experiment I  (n = 5) | Experiment II (n = 25) | | p value |
| --- | --- | --- | --- | --- |
|  |  | Protocol 1  (n = 13) | Protocol 2  (n = 12) |  |
| Weight, kg | 39.6 ± 2.1 | 39.6 ± 1.5 | 39.4 ± 1.7 | 0.967 |
| Systolic arterial pressure, mmHg | 96.6 ± 19.5 | 99.4 ± 24.2 | 91.2 ± 14.5 | 0.565 |
| Diastolic arterial pressure, mmHg | 64.4 ± 16.7 | 65.2 ± 11.7 | 67.3 ± 17.5 | 0.760 |
| CBF, mL/min | 286.2 ± 119.0 | 503.0 ± 414.1 | 381.1 ± 307.4 | 0.519 |
| ETCO_2_, mmHg | 27.1 ± 2.7 | 24.4 ± 3.8 | 27.5 ± 4.3 | 0.206 |
| RVPes, mmHg | 45.2 ± 19.8 | 30.3 ± 9.84 | 31.2 ± 8.9 | 0.197 |
| RVPed, mmHg | 8.5 ± 3.7 | 6.0 ± 4.6 | 5.7 ± 4.2 | 0.478 |
| RVVes, mL | 210 ± 26.1 | 187.2 ± 28.0 | 203.2 ± 38.2 | 0.200 |
| RVVed, mL | 180.2 ± 26.5 | 161.1 ± 29.3 | 170.3 ± 31.8 | 0.481 |
| SV, mL | 38.4 ± 5.1 | 39.1 ± 5.7 | 48.8 ± 26.8 | 0.888 |
| pH | 7.50 ± 0.05 | 7.50 ± 0.04 | 7.47 ± 0.04 | 0.399 |
| SaO_2_, % | 98.8 ± 0.4 | 98.6 ± 0.9 | 98.0 ± 1.0 | 0.109 |
| PaO_2_, mmHg | 126.4 ± 19.6 | 113.7 ± 21.8 | 102.1 ± 17.6 | 0.069 |
| PaCO_2_, mmHg | 37.5 ± 6.4 | 37.1 ± 4.1 | 40.1 ± 4.3 | 0.235 |
| Bicarbonate, mmol/L | 29.1 ± 3.7 | 28.8 ± 2.13 | 29.6 ± 1.9 | 0.360 |
| Lactate, mmol/L | 2.39 ± 0.50 | 1.99 ± 0.57 | 2.26 ± 0.61 | 0.384 |
| Troponin I, ng/mL | 0.050 ± 0.042 | 0.051 ± 0.046 | 0.061 ± 0.075 | 0.925 |

CBF, carotid blood flow; ETCO_2_, end-tidal carbon dioxide; RVPes, end-systolic right ventricular pressure; RVPed, end-diastolic right ventricular pressure; RVVes, end-systolic right ventricular volume; RVVed, end-diastolic right ventricular volume; SV, right ventricular stroke volume; SaO_2_, arterial oxygen saturation; PaO_2_, partial pressure of oxygen; PaCO_2_, partial pressure of carbon dioxide.
