# Supplementary material for: Right ventricular pressure–volume relations and effects of selective vena cava occlusion during cardiopulmonary resuscitation
Source: PLoS One. 2025 Sep 26;20(9):e0333122. doi: 10.1371/journal.pone.0333122 (PMC12469095; doi:10.1371/journal.pone.0333122)
Supplement: S2 Table — (DOCX) [file pone.0333122.s008.docx]

**S2 Table. Comparison of RV PV loop characteristics between interventions according to the time elapsed from CPR initiation.**

|  | **Intervention** | **Spontaneous**  **circulation** | **Time elapsed after CPR initiation** | | | | |
| --- | --- | --- | --- | --- | --- | --- | --- |
|  |  |  | **2 min** | **4 min** | **12 min** | **20 min** | **26 min** |
| Loop eccentricity | **No-VCO** | 1.49 | 0.40 | 0.23 | 0.20 | 0.23 | 0.26 |
|  | **SVCO** | 1.29 | 0.24 | 0.29 | 0.32 | 0.39 | 0.36 |
|  | **IVCO** | 1.52 | 0.24 | 0.26 | 0.32 | 0.36 | 0.34 |
| ESPVR slope | **No-VCO** | 0.18 | 0.56 | 0.73 | 0.90 | 0.80 | 0.81 |
|  | **SVCO** | 0.20 | 0.83 | 0.73 | 0.65 | 0.51 | 0.46 |
|  | **IVCO** | 0.20 | 0.91 | 0.72 | 0.56 | 0.48 | 0.49 |
| EDPVR slope | **No-VCO** | 0.05 | 0.07 | 0.08 | 0.10 | 0.08 | 0.08 |
|  | **SVCO** | 0.03 | 0.07 | 0.07 | 0.07 | 0.07 | 0.06 |
|  | **IVCO** | 0.03 | 0.08 | 0.06 | 0.04 | 0.04 | 0.06 |

CPR, cardiopulmonary resuscitation; VCO, vena cava occlusion; SVCO, superior vena cava occlusion; IVCO, inferior vena cava occlusion; ESPVR, end-systolic pressure–volume relationship; EDPVR, end-diastolic pressure–volume relationship

Loop eccentricity = (RVPes - RVPed) / (RVVed - RVVes); a higher value of loop eccentricity indicates a more elongated loop, reflecting changes in RV loading conditions or systolic/diastolic imbalance.

ESPVR= RVPes / (RVVes - V_0_), it reflects RV contractility, relatively independent of preload and afterload.

EDPVR= RVPed / RVVed, it represents diastolic stiffness (ventricular compliance). A higher value of EDPVR indicates reduced compliance and impaired diastolic relaxation.
