# Supplementary material for: Right ventricular pressure–volume relations and effects of selective vena cava occlusion during cardiopulmonary resuscitation
Source: PLoS One. 2025 Sep 26;20(9):e0333122. doi: 10.1371/journal.pone.0333122 (PMC12469095; doi:10.1371/journal.pone.0333122)
Supplement: S3 Table — (DOCX) [file pone.0333122.s009.docx]

**S3 Table. Comparison of hemodynamic measurements between interventions according to the time elapsed from initiation of CPR.**

|  | **Intervention** | **Spontaneous**  **circulation** | **Time elapsed after CPR initiation** | | | | | **p value** |
| --- | --- | --- | --- | --- | --- | --- | --- | --- |
|  |  |  | **2 min** | **4 min** | **12 min** | **20 min** | **26 min** |  |
| Systolic arterial pressure, mmHg | **No-VCO** | 108.2 ± 15.3 | 81.4 ± 7.9 | 81.4 ± 10.0 | 79.3 ± 12.6 | 65.3 ± 21.1 | 55.6 ± 22.3 | 0.236 |
|  | **SVCO** | 97.6 ± 24.2 | 85.2 ± 18 | 72.8 ± 14.3 | 62 ± 20.9 | 46.3 ± 18.1 | 41.8 ± 16.0 | 0.002 |
|  | **IVCO** | 90.7 ± 14.0 | 90.1 ± 29.1 | 76.1 ± 27.2 | 56.1 ± 18.5 | 47.0 ± 18.6 | 42.6 ± 12.1 | 0.008 |
| Diastolic arterial pressure, mmHg | **No-VCO** | 72.7 ± 16.6 | 23.0 ± 2.7 | 19.9 ± 4.4 | 22.0 ± 7.1 | 14.8 ± 9.0 | 13.7 ± 8.7 | 0.290 |
|  | **SVCO** | 63.9 ± 12.2 | 17.1 ± 7.4 | 17.2 ± 7.5 | 17.1 ± 8.1 | 14.7 ± 6.7 | 16.1 ± 9.3 | 0.641 |
|  | **IVCO** | 66.4 ± 17.1 | 29.9 ± 24.8 | 28 ± 27.4 | 22.4 ± 12.5 | 21.0 ± 14.4 | 19.1 ± 11.9 | 0.645 |
| Mean arterial pressure, mmHg | **No-VCO** | 84.5 ± 13.0 | 42.5 ± 3.2 | 40.4 ± 4.8 | 41.1 ± 8.4 | 31.6 ± 7.8 | 27.7 ± 10.1 | 0.106 |
|  | **SVCO** | 75.1 ± 14.3 | 39.8 ± 8.0 | 35.7 ± 7.0 | 32.1 ± 11.5 | 25.3 ± 10.0 | 26.0 ± 9.6 | 0.030 |
|  | **IVCO** | 72.6 ± 10.8 | 48.2 ± 22.4 | 42.4 ± 24.8 | 32.5 ± 11.3 | 28.8 ± 15.0 | 28.2 ± 11.2 | 0.174 |
| PR, bpm | **No-VCO** | 75.9 ± 58.0 | 101.8 ± 0.1 | 101.8 ± 0.3 | 101.9 ± 0.2 | 101.7 ± 0.2 | 101.8 ± 0.1 | 0.622 |
|  | **SVCO** | 85.6 ± 45.4 | 103 ± 4.4 | 118.4 ± 59.8 | 101.8 ± 0.1 | 102.9 ± 4.1 | 101.8 ± 0.1 | 0.403 |
|  | **IVCO** | 104.1 ± 64.5 | 110.2 ± 29 | 106.1 ± 14.8 | 101.8 ± 0.2 | 101.8 ± 0.2 | 101.7 ± 0.1 | 0.380 |
| CBF, mL/min | **No-VCO** | 365.6 ± 65.3 | 117.6 ± 42.3 | 96.5 ± 30.9 | 73.2 ± 38.8 | 59.5 ± 26.9 | 49.3 ± 5.5 | 0.232 |
|  | **SVCO** | 477.6 ± 409.0 | 154.8 ± 122.2 | 130.2 ± 112.1 | 87.0 ± 98.5 | 43.4 ± 53.9 | 46.1 ± 55.7 | 0.067 |
|  | **IVCO** | 366.2 ± 299.2 | 138.9 ± 104.7 | 110.4 ± 107.9 | 92.8 ± 122.7 | 85.7 ± 123.3 | 83.7 ± 129.8 | 0.871 |
| ETCO_2_, mmHg | **No-VCO** | 27.8 ± 3.3 | 18.1 ± 4.4 | 17.7 ± 5.2 | 18.4 ± 4.3 | 18.6 ± 5.0 | 19.1 ± 8.9 | 0.952 |
|  | **SVCO** | 24.6 ± 3.7 | 14.0 ± 4.7 | 12.6 ± 6.3 | 12.9 ± 5 | 12.4 ± 7.7 | 9.9 ± 6.5 | 0.974 |
|  | **IVCO** | 27.3 ± 4.1 | 10.8 ± 2.2 | 11.1 ± 2.8 | 11.5 ± 4.7 | 9.2 ± 4.4 | 9.2 ± 4.5 | 0.356 |
| RVPes, mmHg | **No-VCO** | 31.4 ± 6.7 | 109.9 ± 37.4 | 135.0 ± 63.8 | 156.5 ± 83.5 | 139.8 ± 69.8 | 137.7 ± 54.5 | 0.887 |
|  | **SVCO** | 33.2 ± 14.4 | 160.2 ± 61.5 | 132.1 ± 59.0 | 113.6 ± 54.1 | 84.6 ± 36.9 | 78.7 ± 35.5 | 0.069 |
|  | **IVCO** | 33.5 ± 11.9 | 173.0 ± 61.2 | 130.5 ± 63.5 | 94.9 ± 53.1 | 79.3 ± 46.8 | 82.8 ± 39.6 | 0.079 |
| RVPed, mmHg | **No-VCO** | 9.9 ± 4.4 | 15.7 ± 2.9 | 17.1 ± 2.2 | 19.8 ± 3.6 | 15.2 ± 1.9 | 15.4 ± 1.4 | 0.056 |
|  | **SVCO** | 6.0 ± 4.5 | 15.7 ± 5.5 | 14.5 ± 4.0 | 13.2 ± 3.8 | 12.5 ± 4.7 | 12.2 ± 4.8 | 0.485 |
|  | **IVCO** | 5.8 ± 4.0 | 18.0 ± 14.9 | 12.7 ± 9.8 | 8.5 ± 2.8 | 8.0 ± 2.8 | 11.2 ± 2.7 | 0.129 |
| RVVes, mL | **No-VCO** | 171.7 ± 30.5 | 195.4 ± 27.3 | 184.1 ± 24.9 | 173.9 ± 27.4 | 173.9 ± 27.8 | 170.1 ± 28.9 | 0.552 |
|  | **SVCO** | 164.4 ± 30.7 | 193.1 ± 31.9 | 182.0 ± 33.8 | 175.3 ± 29.2 | 164.6 ± 44.6 | 170.9 ± 27.3 | 0.231 |
|  | **IVCO** | 171.0 ± 30.6 | 189.7 ± 21.9 | 181.6 ± 21.6 | 168.7 ± 21.3 | 166.9 ± 21.2 | 170.0 ± 28.0 | 0.038 |
| RVVed, mL | **No-VCO** | 203.7 ± 31.1 | 220.8 ± 26.3 | 205.7 ± 29.3 | 196.4 ± 28.2 | 196.9 ± 28.1 | 193.8 ± 31.0 | 0.498 |
|  | **SVCO** | 190.6 ± 29.8 | 218.2 ± 35.0 | 207.8 ± 37.7 | 199.9 ± 35.6 | 185.1 ± 51.0 | 191.1 ± 34.8 | 0.213 |
|  | **IVCO** | 203.2 ± 36.6 | 218.8 ± 28.9 | 205.7 ± 28.9 | 192.0 ± 30.9 | 188.1 ± 28.9 | 190.0 ± 32.8 | 0.056 |
| RV stroke volume, mL | **No-VCO** | 39.3 ± 6.8 | 42.0 ± 5.0 | 30.5 ± 9.2 | 30.0 ± 5.1 | 32.0 ± 8.1 | 34.8 ± 10.4 | 0.054 |
|  | **SVCO** | 39.1 ± 5.5 | 38.3 ± 13.9 | 37.7 ± 18.7 | 36.6 ± 15.4 | 32.6 ± 12.1 | 28.4 ± 12.5 | 0.778 |
|  | **IVCO** | 47.8 ± 25.9 | 41.1 ± 13.6 | 33.4 ± 12.5 | 30.9 ± 13.6 | 28.9 ± 11.4 | 28.1 ± 8.0 | 0.116 |
| SW, mmHg*mL | **No-VCO** | 48.9 ± 33.7 | 818.4 ± 568.6 | 913.1 ± 731.3 | 1187.4 ± 1222.6 | 1010.9 ± 375.7 | 653.9 ± 1180.2 | 0.876 |
|  | **SVCO** | 309.8 ± 267.8 | 1016 ± 856.9 | 1158.4 ± 657.1 | 1005.3 ± 568.3 | 779.2 ± 605.4 | 569.7 ± 571.6 | 0.294 |
|  | **IVCO** | 280.4 ± 254.8 | 1632.3 ± 951.6 | 1067.8 ± 818.1 | 890.5 ± 672.7 | 613.0 ± 501.0 | 687.6 ± 474.5 | 0.266 |
| CO, mL/min | **No-VCO** | 111.2 ± 86.1 | 780.0 ± 572.9 | 876.1 ± 661.1 | 914.3 ± 558.4 | 912.6 ± 811.0 | 789.8 ± 876.1 | 0.995 |
|  | **SVCO** | 553.4 ± 452.0 | 701.3 ± 671.4 | 1075.9 ± 1006.7 | 996.2 ± 758.8 | 874.8 ± 667.3 | 865.0 ± 773.3 | 0.822 |
|  | **IVCO** | 650.8 ± 533.9 | 897.0 ± 689.8 | 844.0 ± 426.6 | 929.7 ± 656.3 | 930.8 ± 682.0 | 1064.2 ± 683.7 | 0.921 |
| RVEF, % | **No-VCO** | 1.0 ± 0.3 | 3.9 ± 2.7 | 4.4 ± 3.0 | 4.9 ± 3.1 | 4.9 ± 3.9 | 4.3 ± 4.3 | 0.965 |
|  | **SVCO** | 4.2 ± 3.6 | 3.5 ± 3.4 | 5.4 ± 4.8 | 5.0 ± 3.2 | 4.6 ± 3.2 | 4.3 ± 3.5 | 0.868 |
|  | **IVCO** | 3.7 ± 2.1 | 4.0 ± 2.4 | 4.2 ± 2.1 | 4.9 ± 3.3 | 5.0 ± 3.3 | 5.7 ± 3.4 | 0.787 |
| Vmax, mL | **No-VCO** | 198.5 ± 35.1 | 208.5 ± 27.0 | 202.2 ± 29.3 | 194.9 ± 28.0 | 196.2 ± 28.8 | 191.3 ± 29.7 | 0.914 |
|  | **SVCO** | 188.7 ± 30.1 | 211.8 ± 35.7 | 205.3 ± 38.2 | 199.5 ± 35.5 | 184.1 ± 53.3 | 190.2 ± 34.7 | 0.438 |
|  | **IVCO** | 199.7 ± 36.3 | 210.9 ± 28.9 | 203.3 ± 28.1 | 189.7 ± 31.1 | 186.3 ± 29.1 | 189.0 ± 32.8 | 0.340 |
| Vmin, mL | **No-VCO** | 177.3 ± 28.7 | 185.6 ± 24.8 | 181.3 ± 24.7 | 173.5 ± 27.1 | 173.3 ± 27.8 | 168.5 ± 27.9 | 0.866 |
|  | **SVCO** | 165.0 ± 31.2 | 187.0 ± 33.9 | 180.4 ± 33.6 | 175.1 ± 28.9 | 163.2 ± 45.9 | 170.3 ± 26.0 | 0.482 |
|  | **IVCO** | 172.3 ± 28.91 | 184.2 ± 20.2 | 180.5 ± 21.3 | 167.4 ± 22.0 | 166.5 ± 21.5 | 169.1 ± 28.0 | 0.221 |
| Pmax, mmHg | **No-VCO** | 31.5 ± 6.6 | 110.0 ± 37.4 | 135.1 ± 63.9 | 156.7 ± 83.6 | 139.8 ± 69.8 | 137.7 ± 54.5 | 0.887 |
|  | **SVCO** | 33.3 ± 14.4 | 160.3 ± 61.5 | 132.4 ± 59.0 | 113.9 ± 54.1 | 84.8 ± 36.9 | 78.8 ± 35.5 | 0.068 |
|  | **IVCO** | 33.6 ± 11.9 | 173.1 ± 61.3 | 130.6 ± 63.5 | 95.0 ± 53.2 | 79.4 ± 46.8 | 83.0 ± 39.7 | 0.079 |
| Pmin, mmHg | **No-VCO** | 5.1 ± 1.2 | 4.5 ± 1.6 | 3.9 ± 2.7 | 4.6 ± 2.1 | 4.0 ± 2.1 | 1.8 ± 4.2 | 0.886 |
|  | **SVCO** | 3.8 ± 2.7 | 4.2 ± 3.1 | 3.6 ± 2.4 | 3.3 ± 2.8 | 3.3 ± 2.4 | 0.5 ± 4.0 | 0.949 |
|  | **IVCO** | 2.6 ± 2.6 | 3.7 ± 3.2 | 3.0 ± 2.2 | 2.9 ± 1.9 | 2.5 ± 1.9 | 0.7 ± 2.9 | 0.836 |
| Pmean, mmHg | **No-VCO** | 15.4 ± 3.0 | 37.4 ± 8.3 | 49.4 ± 22.4 | 55.5 ± 26.0 | 48.7 ± 18.8 | 46.4 ± 13.1 | 0.873 |
|  | **SVCO** | 15.9 ± 6.4 | 53.3 ± 16.8 | 47.6 ± 17.5 | 41.1 ± 15.2 | 32 ± 10.1 | 29.7 ± 10.2 | 0.033 |
|  | **IVCO** | 15.6 ± 4.6 | 57.9 ± 21.7 | 43.8 ± 19.6 | 33.0 ± 12.3 | 28.2 ± 11.3 | 30.1 ± 9.2 | 0.043 |
| Pdev, mmHg | **No-VCO** | 26.3 ± 6.9 | 105.5 ± 38.3 | 131.3 ± 66.4 | 153.6 ± 86.0 | 137.8 ± 69.2 | 135.9 ± 53.0 | 0.889 |
|  | **SVCO** | 30.2 ± 13.3 | 157.8 ± 64.1 | 130 ± 60.7 | 113.0 ± 56.8 | 84.1 ± 39.5 | 78.3 ± 38.1 | 0.098 |
|  | **IVCO** | 31.5 ± 11.7 | 172.8 ± 62.3 | 130.6 ± 64.2 | 95.8 ± 55.3 | 80.4 ± 48.8 | 83.7 ± 41.1 | 0.098 |
| Ea, mmHg/mL | **No-VCO** | 12.5 ± 9.1 | 17.6 ± 9.1 | 2379.6 ± 5266.2 | 24.7 ± 18.9 | 23.4 ± 21.3 | 4.3 ± 47.1 | 0.396 |
|  | **SVCO** | 18.0 ± 46.2 | 40.0 ± 34.9 | 18.4 ± 16.9 | 721.1 ± 2553.3 | 30.9 ± 37.8 | 13.5 ± 19.4 | 0.390 |
|  | **IVCO** | 5.6 ± 4.7 | 106.3 ± 284.5 | 31.1 ± 34.4 | 24.6 ± 33.1 | 11.2 ± 7.0 | 15.7 ± 23.7 | 0.219 |
| PowMax, mmHg*mL/s | **No-VCO** | 16733.3 ± 3806.1 | 57678.0 ± 19895.0 | 58738.0 ± 11328.9 | 63750.0 ± 19330.0 | 68262.0 ± 57659.8 | 67908.0 ± 57707.6 | 0.915 |
|  | **SVCO** | 17913.8 ± 8214.6 | 107933.8 ± 66354.3 | 87933.8 ± 71934.1 | 68636.9 ± 33942.9 | 45174.8 ± 27010.1 | 43106.1 ± 24319.9 | 0.093 |
|  | **IVCO** | 22678.2 ± 12367.7 | 83620.0 ± 36108.1 | 56356.7 ± 34889.6 | 48670.8 ± 42080.7 | 36171.7 ± 24760.6 | 38290.8 ± 25728.1 | 0.366 |
| dP/dt max, mmHg/s | **No-VCO** | 894.0 ± 207.3 | 2387.2 ± 1103.7 | 2374.4 ± 1049.4 | 2740.8 ± 1260.9 | 2455.8 ± 1104.5 | 2631.2 ± 988.6 | 0.869 |
|  | **SVCO** | 808.8 ± 326.4 | 3169.1 ± 1295.3 | 3165.8 ± 2464.0 | 2141.0 ± 1203.3 | 1920.4 ± 1318.6 | 1608.3 ± 659.9 | 0.170 |
|  | **IVCO** | 1432.6 ± 1371.0 | 3229.0 ± 1226.8 | 2624.0 ± 1233.8 | 1921.7 ± 1223.5 | 1725.3 ± 1059.2 | 1715.6 ± 948.0 | 0.160 |
| dP/dt min, mmHg/s | **No-VCO** | -642.5 ± 377.0 | -2098.0 ± 1221.6 | -2233.6 ± 1425.1 | -2572 ± 1586.5 | -1974.2 ± 1189.0 | -2256.0 ± 1168.9 | 0.801 |
|  | **SVCO** | -608.4 ± 213.8 | -2634.0 ± 1384.9 | -2772.2 ± 3065.4 | -1746.4 ± 1048.1 | -1528.0 ± 1394.9 | -1138.7 ± 596.1 | 0.264 |
|  | **IVCO** | -1087.2 ± 1176.7 | -2791.4 ± 1371.2 | -2106.2 ± 1152.4 | -1511.2 ± 1048.0 | -1296.0 ± 927.1 | -1281.1 ± 903.1 | 0.161 |
| dV/dt max, mL/s | **No-VCO** | 771.0 ± 178.2 | 1082.3 ± 255.4 | 1004.1 ± 254.0 | 984.9 ± 178.8 | 1029.9 ± 274.0 | 1009.1 ± 306.4 | 0.957 |
|  | **SVCO** | 925.6 ± 535.1 | 1443.2 ± 593.8 | 1548.2 ± 1084.7 | 1495.0 ± 810.0 | 1189.7 ± 990.1 | 1123.5 ± 817.6 | 0.599 |
|  | **IVCO** | 1121.8 ± 659.4 | 1178.1 ± 372.1 | 933.7 ± 370.3 | 1042.1 ± 562.9 | 943.3 ± 532.5 | 1193.6 ± 680.5 | 0.840 |
| dV/dt min, mL/s | **No-VCO** | -801.8 ± 139.1 | -988.2 ± 103.1 | -958.8 ± 110.2 | -925.7 ± 231.8 | -936.1 ± 275.5 | -901.4 ± 226.3 | 0.970 |
|  | **SVCO** | -1036.5 ± 559.9 | -1429.4 ± 599.1 | -1445.9 ± 1080.2 | -1562.4 ± 961.5 | -1205.9 ± 939.9 | -1158.5 ± 922.2 | 0.652 |
|  | **IVCO** | -1127.1 ± 711.9 | -1121.1 ± 378.4 | -969.7 ± 396.1 | -1052.8 ± 590.5 | -948.2 ± 539.3 | -1185.1 ± 719.5 | 0.872 |
| P@dV/dt max, mmHg | **No-VCO** | 12.7 ± 3.0 | 18.7 ± 5.6 | 17.5 ± 14.4 | 19.0 ± 16.2 | 17.4 ± 18.6 | 32.1 ± 44.6 | 0.985 |
|  | **SVCO** | 12.7 ± 8.2 | 30.2 ± 44.8 | 27.2 ± 39.5 | 21.3 ± 19.9 | 22.2 ± 21.6 | 21.1 ± 18.0 | 0.851 |
|  | **IVCO** | 14.6 ± 6.4 | 12.2 ± 10.2 | 9.3 ± 7.2 | 11.5 ± 9.5 | 10.7 ± 10.2 | 9.1 ± 6.5 | 0.838 |
| P@dP/dt max, mmHg | **No-VCO** | 17.3 ± 3.7 | 72.1 ± 37.5 | 95.2 ± 39.9 | 107.7 ± 72.8 | 98.2 ± 62.3 | 92.7 ± 48.2 | 0.942 |
|  | **SVCO** | 18.6 ± 7.0 | 112.7 ± 36.4 | 91.3 ± 41.5 | 77.2 ± 45.3 | 42.8 ± 28.4 | 40.5 ± 32.1 | 0.010 |
|  | **IVCO** | 19.1 ± 7.9 | 118.2 ± 33.0 | 83.4 ± 46.9 | 60.1 ± 46.3 | 38.8 ± 40.2 | 40.6 ± 34.1 | 0.063 |
| V@dP/dt max, mL | **No-VCO** | 185.3 ± 31.1 | 201.3 ± 26.7 | 195.1 ± 29.4 | 187.9 ± 31.9 | 186.1 ± 30.6 | 180.8 ± 31.5 | 0.887 |
|  | **SVCO** | 176.7 ± 31.4 | 198.8 ± 31.0 | 195.1 ± 35.7 | 188.7 ± 33.0 | 175.8 ± 50.0 | 183.5 ± 31.6 | 0.469 |
|  | **IVCO** | 183.0 ± 30.8 | 195.2 ± 21.4 | 190.8 ± 22.9 | 178.2 ± 25.2 | 177.7 ± 25.0 | 181.4 ± 29.9 | 0.338 |
| V@dP/dt min, mL | **No-VCO** | 188.4 ± 30.0 | 192.1 ± 24.0 | 186.5 ± 28.6 | 180.4 ± 33.3 | 176.9 ± 30.0 | 173.4 ± 31.7 | 0.885 |
|  | **SVCO** | 176.5 ± 30.6 | 194.8 ± 32.9 | 186.2 ± 35.8 | 181.2 ± 30.0 | 168.1 ± 46.3 | 173.7 ± 26.1 | 0.462 |
|  | **IVCO** | 186.9 ± 33.0 | 188.0 ± 19.2 | 183.4 ± 22.4 | 169.2 ± 23.0 | 168.5 ± 22.9 | 170.8 ± 28.4 | 0.210 |
| Tau, ms | **No-VCO** | 48.6 ± 12.1 | 68.0 ± 30.3 | 60.1 ± 37.4 | 66.8 ± 47.1 | 45.6 ± 32.6 | 78.1 ± 47.8 | 0.696 |
|  | **SVCO** | 53.0 ± 21.2 | 76.6 ± 46.4 | 48.6 ± 34.4 | 34.7 ± 19.7 | 35.8 ± 18.9 | 34.7 ± 15.3 | 0.311 |
|  | **IVCO** | 46.6 ± 14.8 | 66.1 ± 39.0 | 50.5 ± 31.3 | 40.4 ± 25.4 | 38.6 ± 19.4 | 43.3 ± 23.8 | 0.486 |

CPR, cardiopulmonary resuscitation; VCO, vena cava occlusion; SVCO, superior vena cava occlusion; IVCO, inferior vena cava occlusion; PR, pulse rate; bpm, beats per minute; CBF, carotid blood flow; ETCO_2_, end-tidal carbon dioxide; RVPes, end-systolic right ventricular pressure; RVPed, end-diastolic right ventricular pressure; RVVes, end-systolic right ventricular volume; RVVed, end-diastolic right ventricular volume; SW, stroke work; CO, cardiac output; RVEF, right ventricular ejection fraction; Vmax, maximal right ventricular volume; Vmin, minimal right ventricular volume; Pmax, maximal right ventricular pressure; Pmin, minimal right ventricular pressure; Pmean, mean right ventricular pressure; Pdev, developed pressure (maximum pressure minus minimal pressure); Ea, elastance (measure of right ventricular afterload); PowMax, maximal power; dP/dt max, maximal value of dP/dt; dP/dt min, minimal value of dP/dt; dV/dt max, maximal value of dV/dt; dV/dt min, minimal value of dV/dt; P@dV/dt max, pressure at maximum of dV/dt; P@dP/dt max, pressure at maximum of dP/dt; V@dP/dt max, volume at maximum of dP/dt; V@dP/dt min, volume at minimum of dP/dt; Tau, relaxation time constant calculated by Weiss method
