# Supplementary material for: Right ventricular pressure–volume relations and effects of selective vena cava occlusion during cardiopulmonary resuscitation
Source: PLoS One. 2025 Sep 26;20(9):e0333122. doi: 10.1371/journal.pone.0333122 (PMC12469095; doi:10.1371/journal.pone.0333122)
Supplement: Attachment — Submitted filename: Responses_to_the_reviewers_s_comments_PLOS one_V3.docx [file pone.0333122.s010.docx]

We thank to the editor and reviewers for their thoughtful recommendations for improving the quality of our manuscript. (Manuscript No. PONE-D-25-22980: Right ventricular pressure-volume relations and effect of selective vena cava occlusion during cardiopulmonary resuscitation) We have revised the manuscript based on the comments and suggestions of the reviewers. We have also provided point-by-point responses to the reviewers’ comments, and the changes made according to the reviewers’ suggestions are shown in red highlights in the enclosed revised manuscript.

Reviewer #1:

Q1) The study was done to explore right ventricular hemodynamic and assess the impact of vena cava occlusion (VCO) on RV pressure-volume during cardiopulmonary resuscitation (CPR). The significance was to see what occurs during relaxation phase of cardiac arrest when vena cava occlusion was applied. It showed that right ventricular end systolic and diastolic significantly decreased with no change in right ventricular volume. This concluded that if venous return is reduced during CPR, then perfusion pressure is affected. Regarding their conclusions, it is well known that one of the causes of cardiac arrest is hypovolemia where there is decrease in venous return. This is managed by giving intravenous fluids and continuing CPR until return of spontaneous circulation is achieved.

The sample size is not large enough as they used 30 pigs with 5 pigs had no vena cava occlusion, but standard CPR, and 25 pigs had vena cava occlusion during CPR. The study did not show if pigs with vena cava occlusion return to spontaneous circulation faster than pigs that did not have vena cava occlusion. If the randomization number was equal, and the study looked at the effect of vena cava occlusion in improving CPR and return of spontaneous circulation during ventricular fibrillation, it will be more beneficial to help improve patient care.

(Our response) This study was conducted to investigate hemodynamics, not to evaluate outcomes, including return of spontaneous circulation. As described in the study's purpose in the Introduction, our experiment was conducted with two objectives. Accordingly, our study consisted of two separate experiments: Experiment I, which assessed right ventricular hemodynamics during standard cardiopulmonary resuscitation (CPR), and Experiment II, which evaluated the impact of vena cava occlusion on right ventricular hemodynamics during CPR. A total of 30 pigs were used in this study, with 5 assigned to Experiment I and 25 to Experiment II. The 5 animals used in Experiment I were not randomized but sequentially selected. The 25 animals used in Experiment II were randomized (N=12 for protocol 1, N=13 for protocol 2) according to the protocol to be applied during the experiment.

We described Experiment I and Experiment II separately in the Method section (Experimental procedures) and revised the manuscript to clarify study designs as follows.

Lines 93-98:

The study was composed of two experiments: Experiment I involved observing RV hemodynamics during standard CPR, whereas Experiment II entailed evaluating the changes in RV hemodynamics after VCO. We used the swine cardiac arrest model with electrically induced ventricular fibrillation (VF). In total, 30 male Yorkshire pigs (35−45 kg) were included. We allocated 5 animals to Experiment I and 25 animals to Experiment II.

Lines 148~176

**Experiment I**

After 2 min of VF, chest compressions with a depth of 5 cm were performed at a rate of 100 compressions/min by using a mechanical CPR device (LUCAS-2® Chest Compression System; Physio-Control, Redmond, WA, USA). Positive-pressure ventilation using room air at a tidal volume of approximately 300 mL was delivered by using a resuscitator bag (Silicone resuscitator 87005133; Laerdal Medical, Stavanger, Norway). Two ventilations were provided per every 30 compressions. CPR was performed for 26 min, and no attempts at restoring spontaneous circulation, such as by defibrillation or epinephrine administration, were made during CPR.

**Experiment II**

After 2 min of VF, chest compressions with a depth of 5 cm were performed at a rate of 100 compressions/min by using a mechanical CPR device (LUCAS-2® Chest Compression System; Physio-Control). Positive-pressure ventilation with room air at a tidal volume of approximately 300 mL was delivered by using a resuscitator bag (Silicone resuscitator 87005133; Laerdal Medical). Two ventilations were provided for every 30 compressions. Hemodynamic effects were evaluated during CPR by alternately occluding and releasing the vena cava for 2-min periods. The sequence of SVC occlusion (SVCO) and IVC occlusion (IVCO) was alternated in the protocols to minimize time-related fluctuations in hemodynamic parameters. The protocol sequences were No-VCO–SVCO–No-VCO–IVCO (2 min each) in Protocol 1 and No-VCO–IVCO–No-VCO–SVCO (2 min each) in Protocol 2. The sequence for each protocol was repeated three times (S2 Fig). Randomization for protocol allocation was performed by using sealed envelopes containing the assigned protocol type; these envelopes were opened by the principal investigator immediately before VF induction. VCO was achieved by filling the balloon of the catheter inserted into the SVC or IVC with 3 mL of normal saline to maintain the balloon diameter at approximately 1 cm. To deflate the balloon to its original state, the saline solution in the balloon was removed. All animals in Experiment II received CPR for approximately 26 min. During CPR, no attempt was made to restore spontaneous circulation, such as by using defibrillation or administering epinephrine.

Q2) The study did not focus on what happened to the ventricular fibrillation when the occlusion was performed and thus it did not show any advantage of performing this procedure during tachyarrhythmias. This should be a major focus as new advances is needed to help bring the patients back to spontaneous circulation. The study did look at the right ventricular pressure volume during the relaxation phase during cardiac arrest and what happens if vena cava occlusion was applied. I don’t see that they have found new data that will improve CPR during cardiac arrest but only minor physiological changes that did not affect those pigs who were in ventricular fibrillation and had CPR performed on them. When I read the article, I was excited to see if the procedure had any influence the arrhythmia. What happened to the pigs that have the occlusion? Did they survive? Did they have a better outcome than the pigs with only standard CPR performed on them. These questions were on my mind, but they were not mentioned. The focus was on the right ventricle and the article concluded that perfusion pressure is affected if venous return is reduced which is obvious and well-known fact.

(Our response) We appreciate the reviewer’s insightful comments and fully share your perspective. To our knowledge, diastolic hemodynamics during cardiopulmonary resuscitation (CPR) have not been previously investigated. Therefore, the primary aim of our study was to explore diastolic right ventricular hemodynamics during CPR, with a particular focus on the contribution of the vena cava to venous return. While we recognize the clinical importance of outcomes such as return of spontaneous circulation or survival, our current study was not designed to evaluate these endpoints, even though we measured important hemodynamic outcomes such as blood pressure and carotid blood flow. Investigating the impact of vena cava occlusion on such outcomes is a critical next step, and we plan to conduct follow-up studies to address this issue. Through this study, we were able to characterize the time-dependent changes in right ventricular pressure-volume loops during CPR and assess how vena cava occlusion affects right ventricular hemodynamics. We believe these findings provide valuable foundational insights for informing future outcome-oriented research. We have revised the manuscript reflecting your valuable comments.

(In the Methods section)

Lines 154~156: CPR was performed for 26 min, and no attempts at restoring spontaneous circulation, such as by defibrillation or epinephrine administration, were made during CPR.

Lines 174~176: All animals in Experiment II received CPR for approximately 26 min. During CPR, no attempt was made to restore spontaneous circulation, such as by using defibrillation or administering epinephrine.

(In the Discussion section)

Lines 384~387: Finally, as this study was designed to investigate RV hemodynamics, we did not assess the effects of VCO on outcomes such as restoration of spontaneous circulation or survival. Further research is warranted to evaluate the impact of VCO on these clinical outcomes.

Reviewer #2:

Q1) The No-VCO group includes only 5 animals, which limits the statistical power for temporal comparisons during standard CPR. This weakens confidence in findings related to time-dependent RV hemodynamic deterioration.

(Our response) Thank you for your comments. This experiment (Experiment I) was designed to observe how right ventricular hemodynamics change over time after CPR from a state of spontaneous circulation. Given that the hemodynamic parameters, including right ventricular pressure-volume loops, are continuously generated with each chest compression (approximately 100 times per minute), we used a minimal number of animals in Experiment I. Additionally, we revised the sample size calculation section by citing a similar study with a comparable experimental design.

Lines 189~193

The sample size was determined with reference to the limited relevant literature available. The sample size for Experiment I was set at five pigs, referring to previous studies that successfully performed PV loop measurements using five animals [12]. For Experiment II, we set the sample size referencing an animal study that assessed left ventricular (LV) end-systolic PV relations with transient IVCO by using an impedance catheter in 24 animals [13].

Q2) VCO periods of only 2 minutes may be too brief to fully manifest hemodynamic effects, as acknowledged by the authors themselves. The rationale for this duration should be discussed in more detail, perhaps informed by prior literature or pilot data.

(Our response) We determined the duration of vena cava occlusion based on a previous animal study, which demonstrated that 10–15 seconds of inferior vena cava occlusion reduced left ventricular systolic pressure by approximately 60 mmHg when evaluating left ventricular pressure-volume relationships. Taking into account that blood flow during cardiopulmonary resuscitation is decreased to about one-third to one-quarter of normal circulation, we extended the occlusion duration to 2 minutes. We added the following paragraph in the Discussion section.

Lines 379~384: Fourth, the duration of VCO may have been insufficient, resulting in minimal hemodynamic effects. In this study, the duration of VCO was based on a previous animal study, which demonstrated that 10–15 s of IVCO reduced LV systolic pressure by approximately 60 mmHg, when evaluating LV PV relationships [13]. Considering that blood flow during CPR is decreased to about one-third to one-quarter of that during normal circulation, we extended the occlusion duration to 2 min [20].

Q3) A key finding—that RV volumes did not significantly change despite reduced RV and systemic pressures during VCO—is counterintuitive. The authors speculate about decreased compliance or short VCO duration, but this remains unresolved. More mechanistic insight or additional measurements (e.g., preload indicators, ventricular elastance) would strengthen this point.

(Our response) As previously discussed, our experimental findings alone could not explain the decrease in right ventricular systolic pressure and systemic arterial pressure without accompanying changes in right ventricular volume during vena cava occlusion. Given the lack of intrinsic cardiac contraction and relaxation, coupled with significant changes in preload and afterload, it is inappropriate to directly apply hemodynamic parameters derived from normal circulation to interpret events during CPR. In response to the reviewers’ suggestions, we have added further descriptions of factors that might have influenced right ventricular volume under these conditions, as follows.

Lines 368~372: During CPR, venous return may occur from other venous compartments or collateral pathways, while the pulmonary compartment and right atrium may act as volume buffers [25,26]. Consequently, significant changes in RVVs might not be apparent. Further research is needed to clarify the mechanisms of blood flow and hemodynamic phenomena during CPR.

Q4) While the study includes several figures and tables, Figure 1–4 and supplemental data are essential to interpreting the results, yet only briefly described. Including more figure-specific interpretation in the main text would help readers grasp the findings.

(Our response) We replenished the descriptions of the figures and supplemental data as follows:

**Fig 1. Representative right ventricular pressure–volume (PV) loops during spontaneous circulation and cardiopulmonary resuscitation (CPR).** The x-axis displays the composite right ventricular volume (mL), and the y-axis shows right ventricular pressure (mmHg). The yellow loop, indicating spontaneous circulation, has a typical elliptical shape. The measurement loops at 2, 4, 12, 20, and 26 min after CPR initiation are shown in blue, green, pink, purple, and red, respectively. During CPR, the PV loops progressively shift into a leftward-leaning trapezoidal shape, indicating the loss of isovolumetric contraction and relaxation, which highlights the passive conduit role of the right ventricle under external chest compressions. Unlike the elliptical loop during spontaneous circulation, these trapezoidal loops exhibit a limited volume range and show a more linear pressure increase during compression, with sharp drops during decompression. These patterns suggest modifications in right ventricular filling and ejection mechanics during CPR.

**Fig 2.** **Changes in right ventricular volumes during spontaneous circulation and during cardiopulmonary resuscitation (CPR).** The right ventricular systolic and diastolic volumes initially increase following the induction of ventricular fibrillation and subsequently gradually decrease during the course of cardiopulmonary resuscitation. RVVed, end-diastolic right ventricular volume; RVVes, end-systolic right ventricular volume; SV, right ventricular stroke volume.

**Fig 3.** **Comparison of right ventricular volumes according to the intervention type**. No significant differences were observed in right ventricular end-systolic and end-diastolic volumes, or in the stroke volume between the No-VCO, SVCO, and IVCO conditions. RVVed, end-diastolic right ventricular volume; RVVes, end-systolic right ventricular volume; SV, right ventricular stroke volume; VCO, vena cava occlusion; SVCO, superior vena cava occlusion; IVCO, inferior vena cava occlusion.

**Fig 4. Representative right ventricular pressure–volume (PV) loops measured during spontaneous circulation and during cardiopulmonary resuscitation (CPR) under different conditions.** No significant differences were observed in right ventricular PV relationships during standard CPR without VCO, with SVCO, and with IVCO**.** (A) PV loops during standard CPR without VCO. The loops show compression-induced pressure fluctuations with reduced stroke volume and a rightward shift as compared to those obtained during spontaneous circulation. (B) PV loops during CPR with SVCO. The overall shape and position of the loops are comparable to those observed during standard CPR without VCO, with no significant changes in stroke volume or pressure development. (C) PV loops during CPR with IVCO. Similar to CPR with SVCO, the loops demonstrate no appreciable difference in right ventricular pressure or volume parameters as compared to standard CPR without VCO. SVCO, superior vena cava occlusion; VCO, vena cava occlusion; IVCO, inferior vena cava occlusion

Q5) Terms like “trapezoidal PV loop” could be better defined or quantified (e.g., loop area, width/height ratio). Figures should also include loop overlays for easier visual comparison.

(Our response) We have added the following references (S3 Fig) to explain RV PV loop shapes. Triangular and trapezoidal PV loops can be described as follows, with relevant content revised:

Lines 49~52:

**Conclusions**

During CPR, the right ventricular pressure–volume loop transforms into a trapezoidal shape, resembling a left-leaning triangle, because the isovolumetric phases disappear. VCO during CPR reduces ventricular systolic pressures, indicating that a reduction in the venous return affects perfusion pressures during resuscitation.

Lines 317~319:

Our observations during Experiment I revealed that, during CPR, the RV PV loop took on a trapezoidal shape, resembling a leftward-leaning triangle, which was distinct from the pattern observed during spontaneous circulation.

Lines 391~394:

This study showed that, during CPR, the RV PV loop transforms into a trapezoidal shape, resembling a leftward-leaning triangle, due to the disappearance of the isovolumetric contraction and relaxation phases. VCO during CPR reduced ventricular systolic pressures, indicating that a reduced venous return affects perfusion pressures during resuscitation.

**S3 Fig (A)	S3 Fig (B)**

**S3 Fig (C)**

**S3 Fig. Right ventricular pressure–volume (PV) loops during cardiopulmonary resuscitation within the first 8 (A), 16 (B), and 24 min (C) in a case.** The measurement loops for the No-VCO, SVCO, and IVCO periods are shown in blue, green, and red, respectively.

VCO, vena cava occlusion; SVCO, superior vena cava occlusion; IVCO, inferior vena cava occlusion.

Q6) This study provides important insights into RV mechanics during CPR and could significantly advance understanding in the field. However, the issues with group size balance, unexplained physiological findings, and underdeveloped interpretation limit its current impact. Addressing these concerns—particularly the unexpected RV volume results and the limited duration of VCO—would considerably enhance the paper's value.

(Our response) We appreciate the reviewer's comments. Despite limited understanding of circulatory physiology during cardiopulmonary resuscitation, our study — the first to directly measure right ventricular pressure-volume loops in this setting — offers meaningful insights into right ventricular hemodynamics and venous return during CPR. These findings may serve as a foundation for future research aimed at optimizing blood flow during resuscitation and improving survival rates. The issue regarding group size balance has been addressed in our response to the previous comment.
