# Supplementary material for: Right ventricular pressure–volume relations and effects of selective vena cava occlusion during cardiopulmonary resuscitation
Source: PLoS One. 2025 Sep 26;20(9):e0333122. doi: 10.1371/journal.pone.0333122 (PMC12469095; doi:10.1371/journal.pone.0333122)
Supplement: Attachment — Submitted filename: response-0902.docx [file pone.0333122.s011.docx]

We thank to the editor and reviewers for their thoughtful recommendations for improving the quality of our manuscript. (Manuscript No. PONE-D-25-22980: Right ventricular pressure-volume relations and effect of selective vena cava occlusion during cardiopulmonary resuscitation) We have revised the manuscript based on the comments and suggestions of the reviewer. We have also provided point-by-point responses to the reviewer’s comments, and the changes made according to the reviewer’s suggestions are shown in red highlights in the enclosed revised manuscript.

**Reviewer #2:**

**This manuscript presents a well-executed experimental study evaluating right ventricular (RV) hemodynamics and the impact of superior and inferior vena cava occlusion (VCO) during cardiopulmonary resuscitation (CPR) using a swine model. The topic is relevant and underexplored, especially in regard to diastolic physiology and RV pressure-volume (PV) dynamics during cardiac arrest. The study introduces novel insights into RV mechanics under CPR conditions and may lay the groundwork for improved resuscitation strategies. However, there are several critical issues regarding design rationale, statistical power, mechanistic interpretation, and clinical applicability that should be addressed or acknowledged more thoroughly.**

**COMMENTS**

**Q1) Consider discussing more directly how the observed hemodynamic changes could theoretically affect CPR success or guide future therapeutic approaches.**

(Our response) We added the following sentences in the Discussion section per the reviewer’s suggestion.

The present study demonstrated a tendency of progressive reduction in the RVVs and SV with an increasing CPR duration. This observation suggested that venous return progressively declines during ongoing resuscitation. Such hemodynamic deterioration may, at least in part, account for the decreased likelihood of successful resuscitation with prolonged CPR. Interventions aimed at augmenting venous return, such as timely administration of intravenous fluid, may mitigate the decline in SV and improve the likelihood of successful resuscitation. Future studies, particularly in clinical settings, are warranted to determine the optimal fluid strategy during prolonged resuscitation and to assess its impact on both short- and long-term outcomes.

**Q2) Include a clearer justification of sample size based on power calculations, variability estimates, or effect size assumptions.**

(Our response) To our knowledge, diastolic hemodynamics during CPR have not been previously investigated. Due to a lack of references, we conducted a few preliminary experiments, which confirmed a small difference in the values of RVV among the VCO periods. Based on these results, we have added details on the sample size calculations for which we received statistical advice as follows.

The sample size was determined with reference to the limited relevant literature available. The sample size for Experiment I was set at five pigs, referring to previous studies that successfully performed PV loop measurements using five animals [12]. Based on two pilot experiments for Experiment II, we extracted preliminary data regarding the difference in RVVs. The sample size was calculated based on the difference in RVV measured at 2 min after the SVCO or IVCO period compared to that measured during standard CPR. Assuming that at least two values were secured for each sector and at least six sample RVVs were extracted per one experiment, the sufficient sample size was determined by determining the difference in the overall RVV mean and standard deviation. A sample size of 25 could achieve 70% power to detect a difference of -3.7 between the null hypothesis mean of zero and the alternative hypothesis mean of 3.7, with a known standard deviation of 18.2 and with a significance level (alpha) of 0.05 based on a two-sided, one-sample *t*-test. We also set the sample size referencing an animal study that assessed left ventricular end-systolic PV relations with transient IVCO by using an impedance catheter in 24 animals [13].

**Q3) This is a key physiological paradox. A deeper mechanistic exploration—possibly through preload surrogates, echocardiography, or vascular resistance estimates—would enhance the scientific contribution. Acknowledge this limitation more explicitly, and suggest longer VCO durations or real-time preload/afterload tracking in future studies.**

(Our response) As you noted, deeper mechanistic exploration, including preload surrogates (CVP, PAOP, RAP, LVEDP), echocardiography (volume, Doppler profiles, IVC diameter, and collapsibility), or vascular resistance estimates (SVR, PVR, Doppler-derived SVR), would be helpful in understanding hemodynamics, including the mechanisms of venous return, during CPR. Furthermore, future studies are needed to assess the hemodynamic impact of longer VCO and to introduce tools capable of real-time preload/afterload tracking. However, because precise hemodynamic measurements are difficult to obtain during CPR, new methodologies beyond PV loop measurements will be necessary for precise measurements. We added the following sentence to the limitations of the Discussion.

Finally, further mechanistic exploration, including preload surrogates, echocardiography, or estimates of vascular resistance, would facilitate an understanding of the hemodynamics of venous return during CPR. Future studies are needed to assess the hemodynamic impact of longer periods of VCO and to introduce tools capable of real-time preload/afterload tracking. However, as precise hemodynamic measurements are difficult to obtain during CPR, new methodology beyond PV loop measurements will be necessary.

**Q4) Include a table summarizing key geometric PV loop characteristics or define shape transformation metrics quantitatively. Loop overlays for visual comparison would enhance clarity.**

(Our response) Based on your feedback, we've created new figures and tables (S3 and S4 figures, S3 table). And, we added characteristics of PV loop summarized sentence in the Result section.

The loop overlay extracts representative loops for each 2-minute period and describes them as follows (S3 Figures). Compared to the spontaneous circulation period marked in black, when CPR is performed with No-VCO, RVVed and RVVes increase, but the effective volume (SV) does not seem to increase significantly. In addition, no significant differences were observed in the RV stroke volume between the No-VCO (blue), SVCO (green), and IVCO (red) conditions.

In addition, S4 figures contain analysis of loop eccentricity and pressure-volume relationship slope (ESPVR and EDPVR). We believe these graphs will help understand changes in the RV PV loop during CPR.

**Effects of VCO during CPR on hemodynamics**

…The loop eccentricity declined over time, reflecting that the loop became relatively wider horizontally. The end-systolic pressure–volume relationship slope, reflecting RV contractility, gradually decreased over the course of CPR in all three groups, with the lowest value occurring in the period of spontaneous circulation. The end-diastolic pressure–volume relationship slope, reflecting ventricular compliance, remained low overall and showed only modest changes over time and across groups (S3 Table, S4 Fig).

**S3 Fig. RV PV loops during CPR within the first 8 (A), 16 (B), and 24 min (C) in a representative case.** Measurement loops for the No-VCO, SVCO, and IVCO periods are shown in blue, green, and red, respectively. Compared to the spontaneous circulation period, marked in black, when CPR is performed with No-VCO, both RVVed and RVVes increase, but the effective volume does not seem to increase significantly. In addition, no significant differences were observed in the RV stroke volume between the No-VCO, SVCO, and IVCO conditions.

RV PV, right ventricular pressure–volume; CPR, cardiopulmonary resuscitation; VCO, vena cava occlusion; SVCO, superior vena cava occlusion; IVCO, inferior vena cava occlusion; RVVed, end-diastolic right ventricular volume; RVVes, end-systolic right ventricular volume

**S3 Fig (A)**

**S3 Fig (B)**

**S3 Fig (C)**

**S3 Table. Comparison of RV PV loop characteristics between interventions according to the time elapsed from CPR initiation.**

|  | **Intervention** | **Spontaneous**  **circulation** | **Time elapsed after CPR initiation** | | | | |
| --- | --- | --- | --- | --- | --- | --- | --- |
|  |  |  | **2 min** | **4 min** | **12 min** | **20 min** | **26 min** |
| Loop eccentricity | **No-VCO** | 1.49 | 0.40 | 0.23 | 0.20 | 0.23 | 0.26 |
|  | **SVCO** | 1.29 | 0.24 | 0.29 | 0.32 | 0.39 | 0.36 |
|  | **IVCO** | 1.52 | 0.24 | 0.26 | 0.32 | 0.36 | 0.34 |
| ESPVR slope | **No-VCO** | 0.18 | 0.56 | 0.73 | 0.90 | 0.80 | 0.81 |
|  | **SVCO** | 0.20 | 0.83 | 0.73 | 0.65 | 0.51 | 0.46 |
|  | **IVCO** | 0.20 | 0.91 | 0.72 | 0.56 | 0.48 | 0.49 |
| EDPVR slope | **No-VCO** | 0.05 | 0.07 | 0.08 | 0.10 | 0.08 | 0.08 |
|  | **SVCO** | 0.03 | 0.07 | 0.07 | 0.07 | 0.07 | 0.06 |
|  | **IVCO** | 0.03 | 0.08 | 0.06 | 0.04 | 0.04 | 0.06 |

CPR, cardiopulmonary resuscitation; VCO, vena cava occlusion; SVCO, superior vena cava occlusion; IVCO, inferior vena cava occlusion; ESPVR, end-systolic pressure–volume relationship; EDPVR, end-diastolic pressure–volume relationship

Loop eccentricity = (RVPes - RVPed) / (RVVed - RVVes); a higher value of loop eccentricity indicates a more elongated loop, reflecting changes in RV loading conditions or systolic/diastolic imbalance.

ESPVR= RVPes / (RVVes - V_0_), it reflects RV contractility, relatively independent of preload and afterload.

EDPVR= RVPed / RVVed, it represents diastolic stiffness (ventricular compliance). A higher value of EDPVR indicates reduced compliance and impaired diastolic relaxation.

**S4 Fig (A). RV PV loop eccentricity during CPR.** Measurement loops for the No-VCO, SVCO, and IVCO periods are shown in yellow, blue, and green, respectively. Loop eccentricity was calculated as (RVPes - RVPed) / (RVVed - RVVes), where higher values indicates a more elongated loop, reflecting changes in RV loading conditions or systolic/diastolic imbalance. The eccentricity decreases over time, reflecting that the loop became relatively wider horizontally (greater volumetric than pressure fluctuations).

RV PV, right ventricular pressure–volume; VCO, vena cava occlusion; SVCO, superior vena cava occlusion; IVCO, inferior vena cava occlusion; SV, right ventricular stroke volume; Pedv, developed pressure (maximum pressure minus minimal pressure); Spont; spontaneous circulation period

**S4 Fig (B). End-systolic pressure–volume loop relationship slope during CPR.** ESPVR was calculated as RVPes / (RVVes - V_0_) and was used as an index of RV contractility. The ESPVR slope gradually decreased (decreased contractility) over the course of CPR in all three groups, with the lowest value occurring in the period of spontaneous circulation.

ESPVR, end-systolic pressure–volume relationship; VCO, vena cava occlusion; SVCO, superior vena cava occlusion; IVCO, inferior vena cava occlusion; RVPes, end-systolic right ventricular pressure; ESV, end-systolic right ventricular volume; Spont; spontaneous circulation period

**S4 Fig (C). End-diastolic pressure–volume loop relationship slope during CPR.** EDPVR was defined as RVPed / RVVed and was used as an index of diastolic stiffness, with higher values indicating reduced compliance. The EDPVR slope remains low overall and shows only modest changes over time and across groups.

EDPVR, end-diastolic pressure–volume relationship; VCO, vena cava occlusion; SVCO, superior vena cava occlusion; IVCO, inferior vena cava occlusion; RVPed, end-diastolic right ventricular pressure; EDV, end-diastolic right ventricular volume; Spont; spontaneous circulation period

**Q5) Use consistent definitions of acronyms (e.g., RVVes, RVVed) throughout text and figures to reduce reader confusion.**

(Our response) Thank you for your comment. Some words in figures, tables and supplemental figures/tables were changed to acronyms as below. (CPR, PV, RV, RVV, RVP, VF, RVVes, RVVed, SV, SVC, IVC)

**Fig 1. Representative RV PV loops during spontaneous circulation and during CPR.** The x-axis displays the composite RVV (mL), and the y-axis shows the RVP (mmHg). The yellow loop, indicating spontaneous circulation, has a typical elliptical shape. The measurement loops at 2, 4, 12, 20, and 26 min after CPR initiation are shown in blue, green, pink, purple, and red, respectively. During CPR, the PV loops progressively take on a trapezoidal shape, indicating the loss of isovolumetric contraction and relaxation. This highlights the passive conduit role of the RV under external chest compressions. Unlike the elliptical loop during spontaneous circulation, these trapezoidal loops exhibit a limited volume range and show a more linear pressure increase during compression, with sharp drops during decompression. These patterns suggest modifications in RV filling and ejection mechanics during CPR.

RV, right ventricular; PV, pressure–volume; CPR, cardiopulmonary resuscitation; RVV, right ventricular volume; RVP, right ventricular pressure

**Table 1. Hemodynamic measurement at baseline and during CPR in the No-VCO group**

.. SV, right ventricular stroke volume

**Fig 2.** **Changes in RVVs during spontaneous circulation and during CPR.** The right ventricular systolic and diastolic volumes initially increase following the induction of VF and subsequently gradually decrease during the course of CPR.

RVVs, right ventricular volumes; CPR, cardiopulmonary resuscitation; VF, ventricular fibrillation; RVVed, end-diastolic right ventricular volume; RVVes, end-systolic right ventricular volume; SV, right ventricular stroke volume

**Fig 3.** **Comparison of RVVs according to the intervention type**. No significant differences were observed in RVVed and RVVes, or in the SV between the No-VCO, SVCO, and IVCO conditions.

RVVs, right ventricular volumes; RVVed, end-diastolic right ventricular volume; RVVes, end-systolic right ventricular volume; SV, right ventricular stroke volume; VCO, vena cava occlusion; SVCO, superior vena cava occlusion; IVCO, inferior vena cava occlusion

**Table 2. Comparisons of hemodynamic values between groups.**

… SV, right ventricular stroke volume

**Fig 4. Representative RV PV loops measured during spontaneous circulation and during CPR under different conditions.** No significant differences were observed in RV PV relationships during standard CPR without VCO, with SVCO, and with IVCO**.** (A) PV loops during standard CPR without VCO. The loops show compression-induced pressure fluctuations with reduced SV and a rightward shift as compared to those obtained during spontaneous circulation. (B) PV loops during CPR with SVCO. The overall shape and position of the loops are comparable to those observed during standard CPR without VCO, with no significant changes in SV or pressure development. (C) PV loops during CPR with IVCO. Similar to CPR with SVCO, the loops demonstrate no appreciable difference in RVP or RVV parameters as compared to standard CPR without VCO.

RV PV, right ventricular pressure–volume; CPR, cardiopulmonary resuscitation; VCO, vena cava occlusion; SVCO, superior vena cava occlusion; IVCO, inferior vena cava occlusion; SV, right ventricular stroke volume; RVP, right ventricular pressure; RVV, right ventricular volume

**S1 Fig. Confirmation of catheter placement.** Chest anteroposterior X-ray image showing catheter placement. The image was obtained using a portable chest X-ray device. The red arrow indicates a PV catheter in the right ventricle. The blue arrows indicate the balloon catheters in the proximal portion of the SVC and IVC.

PV, pressure–volume; SVC, superior vena cava; IVC, inferior vena cava

**S3 Fig. RV PV loops during CPR within the first 8 (A), 16 (B), and 24 min (C) in a representative case.** Measurement loops for the No-VCO, SVCO, and IVCO periods are shown in blue, green, and red, respectively. Compared to the spontaneous circulation period, marked in black, when CPR is performed with No-VCO, both RVVed and RVVes increase, but the effective volume does not seem to increase significantly. In addition, no significant differences were observed in the RV stroke volume between the No-VCO, SVCO, and IVCO conditions.

RV PV, right ventricular pressure–volume; CPR, cardiopulmonary resuscitation; VCO, vena cava occlusion; SVCO, superior vena cava occlusion; IVCO, inferior vena cava occlusion; RVVed, end-diastolic right ventricular volume; RVVes, end-systolic right ventricular volume

**S1 Table. Baseline characteristics and measurements of animals included in Experiments I and II…**. ; SV, right ventricular stroke volume;

**S2 Table. Comparison of hemodynamic measurements between interventions according to the time elapsed from initiation of CPR…**

CPR, cardiopulmonary resuscitation; VCO, vena cava occlusion; SVCO, superior vena cava occlusion; IVCO, inferior vena cava occlusion; PR, pulse rate; bpm, beats per minute…

**Q6) The manuscript is well written, with high technical clarity. However, some repetitions exist (e.g., "trapezoidal loop resembling a leftward-leaning triangle" appears in several sections).**

(Our response) We have removed the repeated sentences as below, leaving only the phrase ‘a trapezoidal shape, resembling a leftward-leaning triangle’ written in the abstract and conclusion sections.

Discussion

Our observations during Experiment I revealed that, during CPR, the RV PV loop took on a trapezoidal shape, ~~resembling a leftward-leaning triangle~~, which was distinct from the pattern observed during spontaneous circulation. This change could be attributed to the loss of both isovolumetric contraction and relaxation periods. Furthermore, this indicated a physiological change caused by pulmonary hypertension during CPR.

**Fig 1. Representative RV PV loops during spontaneous circulation and CPR…** During CPR, the PV loops progressively take on a trapezoidal shape, indicating the loss of isovolumetric contraction and relaxation. This highlights the passive conduit role of the RV under external chest compressions.
